# Tachycardia-Induced Cardiomyopathy in a Young Healthy Patient: A Case Report

**DOI:** 10.7759/cureus.28932

**Published:** 2022-09-08

**Authors:** Zahid Khan, George Besis, Joseph Tomson

**Affiliations:** 1 Acute Medicine, Mid and South Essex NHS Foundation Trust, Southend-on-Sea, GBR; 2 Cardiology and General Medicine, Barking, Havering and Redbridge University Hospitals NHS Trust, London, GBR; 3 Cardiology, Royal Free Hospital, London, GBR

**Keywords:** heart palpitations, cardiac magnetic resonance (cmr), left ventricular systolic dysfunction, ironman marathon, lone atrial fibrillation, tachycardia induced cardiomyopathy, reversible cardiomyopathy

## Abstract

Tachycardia-induced cardiomyopathy (TIC) can result in both systolic and/or diastolic ventricular dysfunction as a result of the prolonged fast heart rate which is reversible upon controlling the fast heart rate or arrhythmia. The exact heart rate that can lead to this is not clear, however, a heart rate > 100 in general needs attention. Tachycardia-induced cardiomyopathy is a well-established cause of left ventricular dysfunction which usually happens due to an increased atrial or ventricular rate. The incidence of TIC is very low although the exact incidence is unclear. It should be considered in all patients with dilated cardiomyopathy or those with no obvious explanation for dilated cardiomyopathy and in presence of tachycardia or atrial fibrillation with a rapid ventricular response. Tachycardia-induced cardiomyopathy has also been labeled as arrhythmia-induced cardiomyopathy lately. We present a case of a 50-year-old patient who presented with a fever of 39^o^C, feeling generally unwell, had a sore throat, and collapsed at home after several episodes of vomiting after two days of intense exercise. He was diagnosed with suspected tonsillitis and was treated with co-amoxiclav. He was exercising over 10 hours weekly for the last two months in the gym for the Ironman triathlon in London. An echocardiogram showed severe left ventricular systolic dysfunction (LVSD) with a left ventricular ejection fraction (LVEF) of 25%. An electrocardiogram showed sinus tachycardia with a right bundle branch block (RBBB). Cardiac magnetic resonance imaging (CMR) showed normal biventricular function with an ejection fraction (EF) of 71% four months later. The patient was diagnosed with tachycardia-induced cardiomyopathy. This case is unique as the patient presented with transient severe LVSD after training for the ironman triathlon and spontaneous recovery.

## Introduction

Over 800 marathon races are organized worldwide every year and several thousand individuals participate in them every year [1]. Tachycardia-induced cardiomyopathy (TIC) was first described in 1913 in a young patient who presented with heart failure and atrial fibrillation with rapid ventricular response [2]. Whipple et al. described the first experimental TIC in 1962 by demonstrating that rapid and protracted atrial pacing led to cardiac failure with low output and sustained rapid atrial or ventricular pacing caused by severe biventricular systolic and diastolic dysfunction with dilatation of all four chambers in animal models [3,4].

The relationship between tachyarrhythmia and reversible heart failure was first described by Phillips et al. in 1949 [5]. Tachycardia-induced cardiomyopathy has been shown to occur both in experimental models and in patients with tachyarrhythmia. Tachycardia-induced cardiomyopathy may follow atrial or ventricular arrhythmias such as atrial fibrillation, supraventricular tachycardia, and ventricular arrhythmia [6]. The exact incidence of TIC is not clear, and the majority of evidence comes from case reports or case series [7]. Tachycardia-induced cardiomyopathy can happen at any age and has been reported in utero, infants, children, and adults and the commonly associated arrhythmia is atrial fibrillation [8,9,10]. There are two proposed theories with regards to TIC, the first one being that ventricular dysfunction is primarily arrhythmia-induced and the second one in which the arrhythmia exacerbates ventricular dysfunction in a patient with concomitant heart disease (arrhythmia-mediated) [11]. The exact mechanism for TIC is not clear and includes calcium overload, energy metabolism abnormalities, redox stress, and subclinical ischemia [11].

It is a well-known fact that chronic tachycardia can result in structural heart changes including left ventricular dilatation and cellular morphological changes [12]. These changes may result in marked depression of left ventricular ejection fraction (LVEF), elevated filling pressures, depressed cardiac output, and increased systemic vascular resistance and although in most cases these changes are reversible, in certain cases the LVEF may not return to a normal level [13-16]. Chronic tachycardia that persists for 10% to 15% of the days has been suggested to be associated with cardiomyopathy and as mentioned earlier, there is no fixed heart rate for this association although any heart rate > 100 has generally been associated with this condition [17]. We present a case of a 50-year-old male who presented with collapse at home after intense training for the ironman triathlon for the past few weeks and was diagnosed with TIC. The patient made a complete recovery indicating the reversibility of this condition.

## Case presentation

**Figure 1:**
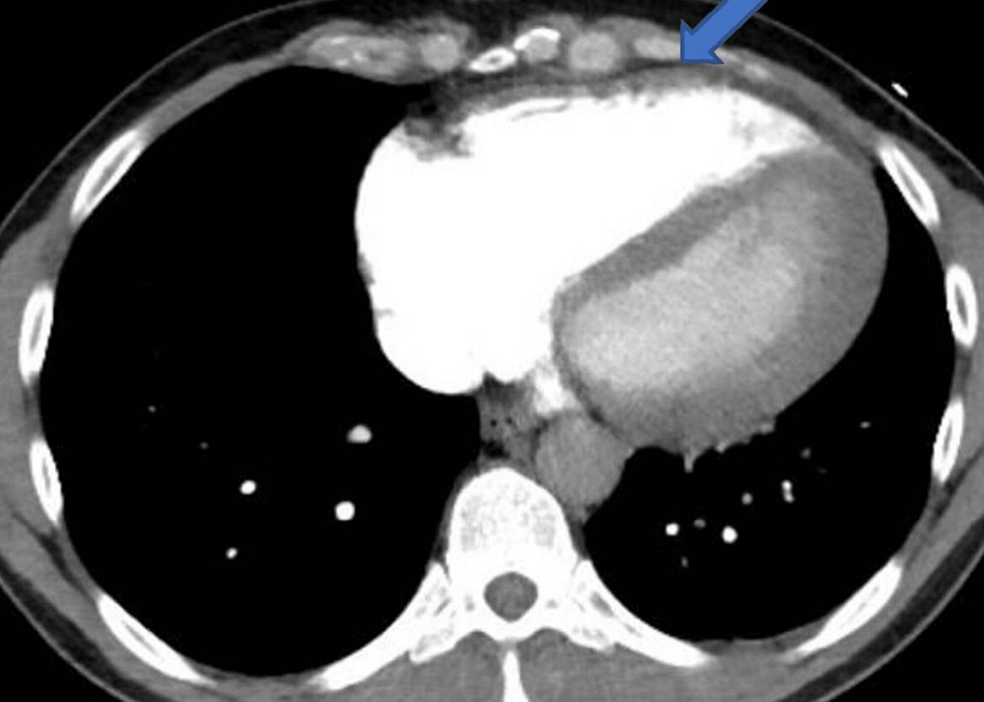
Computerized tomography angiogram shows right ventricular enlargement (blue arrow)

A 50-year-old male presented with fever, sore throat, and vomiting several times followed by collapse at home after two days of intense exercise. Past medical history (PMH) was significant with hypertension, and dyslipidemia. Regular medications include felodipine and atorvastatin. He was preparing for the London ironman triathlon and was exercising over 10 hours every week for the last two months which included heavy lifting at the gym and cardio exercises. He started to feel unwell two days prior to hospital admission. He was commenced on oral amoxicillin by his general practitioner for possible tonsillitis. Vitals on arrival were as follows: temperature of 37.1 °C, heart rate (HR) 74 respiratory rate (RR) 20, blood pressure (BP) 99/63, a saturation of peripheral oxygen (SpO2) at 96%, and weight at 68 kg. Clinical examination was unremarkable apart from mild tonsillar erythema. His chest was clear, heart sounds were normal, and he did not have any pedal edema. The electrocardiogram (ECG) showed sinus tachycardia (HR > 110) with a right bundle branch block (RBBB). Laboratory results showed only elevated serum urea 8.9 mmol/L (normal range: 2.1-7.1), total cholesterol 7.0 mmol/L (0-5), troponin were 12 and 13 (normal <14 ng/l). Computerized pulmonary angiography showed right ventricular enlargement with right ventricle (RV):left ventricle (LV) ratio 1:1 but no pulmonary embolism (Figure 1).

Echocardiography showed non-dilated LV with septal hypertrophy and severely impaired LV systolic function (LVEF: 25%). The septum was dyskinetic and RV was severely dilated (Videos 1-3).

**Video 1:** Echocardiogram apical four-chamber view shows impaired left ventricular systolic function and dilated right ventricle

**Video 2:** Echocardiogram PSAX view shows impaired LVSF PSAX: Parasternal short axis view, LVSF: Left ventricular systolic function

**Video 3:** Echocardiogram PLAX shows impaired LVSF PLAX: Parasternal long axis view, LVSF: Left ventricular systolic function

The patient was not commenced on any rate-limiting medications due to hypotension and a repeat echocardiogram showed significant improvement in LVEF up to 45% within 48 hours without any obvious treatment (Videos 4-6).

**Video 4:** Echocardiogram PSAX view shows mild LVSD PSAX: Parasternal short axis view, LVSD: Left ventricular systolic dysfunction

**Video 5:** Echocardiogram apical four-chamber view shows marked improvement in left ventricular systolic function within 48 hours

**Video 6:** Echocardiogram apical four-chamber view shows improved left ventricular function and moderately dilated right atrium

The patient remained in normal sinus rhythm and did not have any further tachycardia. He was discharged home only on his regulation medications and an outpatient follow-up was arranged. He was advised to avoid intense exercises given his TIC. The patient was seen at the outpatient heart failure and arrhythmia clinic three months after discharge. Cardiac magnetic resonance (CMR) imaging showed normal LVSF, ejection fraction (EF) was 71% with no evidence of regional wall motion abnormality (RWMA) or mucosal edema, and moderate right atrial enlargement. The patient was advised against intense exercises such as marathons and to continue with his regular medications.

The possible differential diagnosis, in this case, includes myocardial infarction, myocarditis, hypertrophic cardiomyopathy, and myocardial bridging. There was no evidence of myocardial infarction (MI) in this patient and the ECG did not show any features of MI. The CMR did not show any evidence of myocarditis or hypertrophic cardiomyopathy and/or myocardial bridging. In addition, his LVEF improved within 48 hours without any treatment which is highly suggestive of TIC.

## Discussion

Tachycardia-induced cardiomyopathy is reversible cardiomyopathy with left ventricular systolic dysfunction that can be induced by any tachyarrhythmia and early recognition, and treatment mostly results in left ventricular function recovery [18]. Tachycardia-induced cardiomyopathy was first described in the early 20th century and since then, multiple studies have established a causal relationship between tachycardia and cardiomyopathy [19]. Early recognition of heart failure and tachycardia allows clinicians to optimally treat this condition leading to complete recovery of the left ventricular function in most patients [18]. It can be caused by various tachyarrhythmias including sinus tachycardia, atrial fibrillation, atrial tachycardia, supraventricular tachycardia, and ventricular tachycardia [18,19]. Tachycardia-induced cardiomyopathy is poorly understood in reality and the likely cause for this is that tachyarrhythmia is considered to be a consequence of cardiomyopathy rather than tachyarrhythmia being a cause of cardiomyopathy [20]. The duration from the onset of tachyarrhythmias leading to cardiomyopathy depends on the type of tachyarrhythmia, comorbidities, and any underlying structural heart disease [17,20].

The first case of TIC was discussed in 1913 in a young patient who presented with congestive heart failure and atrial fibrillation with rapid ventricular response (AFRVR) [2,20]. Previous studies have shown that LVSF resolves in about four weeks once heart rate is appropriately controlled [20]. A retrospective study based on a comparison of matched TIC and non-TIC dilated cardiomyopathy patients showed that a left ventricular diastolic dimension (LVDD) of ≤61mm predicted TIC with a sensitivity of 100% and specificity of 71.4% [20,21]. Tachycardia-induced cardiomyopathy can present at any age and supraventricular arrhythmias are more frequently reported than ventricular arrhythmias and recently frequent ventricular ectopics have been reported to be associated with it [22]. The likely explanation for TIC is the fact that high ventricular rates result in cardiac dilatation and mitral regurgitation which are known to be associated with elevated ventricular filling pressures, decreased contractility, biventricular wall thinning resulting in reduced cardiac output, and increased systemic vascular resistance leading to heart failure with neurohormonal activation [22]. Other possible changes include cellular changes such as loss of myocytes, cellular elongation, myofibril misalignment, and loss of sarcomere register, which could occur due to the derangement of the extracellular matrix [15].

Sudden cardiac death (SCD) has been reported in marathon runners and the overall incidence varies from 1:50,000 to 1:220,000 [23]. The incidence of SCD from marathons is lower in women compared to men and only 15% of deaths due to marathons have been reported in women. However, the overall incidence might not be different because the participation of women in marathons is also lower. The major cause of both fatal and non-fatal cardiac arrests is due to coronary artery disease (CAD) [23]. Multiple studies have reported a rise in troponin T and I following marathons, and the levels have been up in about 30% to 68% of non-elite marathon runners immediately following the race [23]. This number is even higher (86%) when highly sensitive newer generation troponin assays are used and the level drops quickly within 24 hours suggestive of the fact that troponin rise is due to increased membrane permeability rather than permanent myocyte damage and this is supported by the absence of delayed enhancement on CMR imaging [23-24]. Both RV and LV changes have been reported in marathon participants although RV dilatation and decreased RV systolic and diastolic function although these changes return to normal within a week after a marathon [23-24].

Another study showed increased pulmonary artery pressure (PAPs), increased RV dimensions, and decreased RV function, and alterations in LV diastolic function. These changes correlate with troponin release and an increase in N-terminal (NT)-pro brain natriuretic peptide (BNP). These changes were found to be influenced by the participants' level of preparation for the marathon race and the most striking abnormalities were found in participants who trained ≤35 miles/week before the marathon [24-25]. This study also showed that moderate-intensity strenuous cycling for one hour among trained athletes was associated with an almost twofold increase in pulmonary capillary wedge pressure and mean pulmonary artery pressure (mPAP). On the other hand, ultra-marathon runners performing at high altitudes had a transient increase in pulmonary artery pressure (PAP) with RV dilatation and RV dysfunction and they did not have any significant elevation in the less sensitive troponin assay. The authors suggested that the increase in pulmonary pressures was multifactorial and occurred in association with impaired LV relaxation and perhaps an intrinsic increase in pulmonary vascular resistance [25].

Clinical studies have reported improvement in LVSF within a month following the treatment of tachyarrhythmia, however, complete recovery can take two to three months and rarely takes more than six months. Furthermore, this duration can be shortened by adequate heart rate reduction treatment. Another study reported significantly higher LV volumes in patients with TIC when compared to age, sex, and ejection fraction matched controls and the key to treating TIC is to eliminate or control the culprit tachyarrhythmia which can be achieved either through antiarrhythmic medications or radiofrequency catheter ablation (RFCA) [18,25]. Our patient also showed complete recovery of his LVF by controlling his heart rate and avoidance of excessive exercise.

The LVSF returns to normal within two weeks after the rapid pacing stops and hemodynamic parameters return to normal within four weeks although animal models have shown that complete recovery of LV function and LV dilatation may not happen [18]. Studies have shown that long-term exercise training is associated with cardiac remodeling and a J-shaped association was reported to be present between the level of physical activity and atrial fibrillation risk [26]. Similarly, coronary artery atherosclerosis has been reported to be more prevalent in middle-aged athletes than inactive individuals and active athletes have a higher prevalence of heavily calcified stable plaques, however, this has not been reported to be associated with increased cardiovascular events. Furthermore, myocardial fibrosis is also more common in competitive athletes and there is conflicting evidence with regard to the association between high volumes of physical activity and health risk. Some studies have suggested plateauing of the risk reduction at the highest exercise volumes whereas others have described this relationship to be U-shaped with an increase in risk at the highest exercise volumes [26,27]. Athletes performing high-volume and high-intensity exercise have higher coronary artery calcification (CAC) scores with unknown clinical significance and the coronary artery size and dilating capacity are increased among athletes compared to control which could offset the apparently negative adaptation of a higher CAC. Athletes might be at increased cardiovascular disease (CVD) risk due to an elevated CAC score, but this hypothesis is yet to be proved by statistical data. The higher CAC score could be due to exercise-related changes in calcium metabolism and increased parathyroid hormone release in athletes which leads to intra-arterial calcification [26,28].

## Conclusions

In conclusion, our patient presented with TIC after performing strenuous exercises for a few weeks. This condition can present in patients who perform strenuous exercises and in marathon runners. The condition is reversible, and patients show complete recovery with appropriate management of tachyarrhythmia. Most patients show recovery within two to three months and the biventricular function returns to normal. The condition is more commonly caused by supraventricular tachycardia than ventricular tachycardia. The key management strategy is antiarrhythmic medications, trigger avoidance, and rarely radio-frequency catheter ablation.
